# High-dose ascorbic acid synergizes with anti-PD1 in a lymphoma mouse model

**DOI:** 10.1073/pnas.1908158117

**Published:** 2020-01-07

**Authors:** Rebecca A. Luchtel, Tushar Bhagat, Kith Pradhan, William R. Jacobs, Mark Levine, Amit Verma, Niraj Shenoy

**Affiliations:** ^a^Department of Medicine (Oncology), Albert Einstein College of Medicine, Montefiore Medical Center, Bronx, NY 10461;; ^b^Department of Microbiology and Immunology, Albert Einstein College of Medicine, Montefiore Medical Center, Bronx, NY 10461;; ^c^Department of Molecular Genetics, Albert Einstein College of Medicine, Montefiore Medical Center, Bronx, NY 10461;; ^d^Molecular and Clinical Nutrition Section, Intramural Research Program, National Institute of Diabetes and Digestive and Kidney Diseases, National Institutes of Health, Bethesda, MD 20892;; ^e^Experimental Therapeutics Program, Albert Einstein Cancer Center, Albert Einstein College of Medicine, Bronx, NY 10461

**Keywords:** ascorbic acid, vitamin C, immunotherapy, checkpoint inhibition, anti-PD1

## Abstract

Major efforts are underway to identify agents that can potentiate effects of immune checkpoint inhibition. Here, we show that ascorbic acid (AA) treatment caused genomewide demethylation and enhanced expression of endogenous retroviral elements in lymphoma cells. AA also increased 5-hydroxymethylcytosine (5hmC) levels of CD8+ T cells and enhanced their cytotoxic activity in a lymphoma coculture system. High-dose AA treatment synergized with anti-PD1 therapy in a syngeneic lymphoma mouse model, resulting in marked inhibition of tumor growth compared with either agent alone. Analysis of the intratumoral epigenome revealed increased 5hmC with AA treatment, consistent with in vitro findings. Analysis of the tumor immune microenvironment revealed that AA strikingly increased intratumoral infiltration of CD8+ T cells and macrophages, suggesting enhanced tumor immune recognition. The combination treatment markedly enhanced intratumoral infiltration of macrophages and CD8+ T lymphocytes, granzyme B production by cytotoxic cells (cytotoxic T cells and natural killer cells), and interleukin 12 production by antigen-presenting cells compared with single-agent anti-PD1. These data indicate that AA potentiates anti-PD1 checkpoint inhibition through synergistic mechanisms. The study provides compelling rationale for testing combinations of high-dose AA and anti-PD1 agents in patients with aggressive B cell lymphoma as well as in preclinical models of other malignancies.

## Significance

New strategies are needed to improve efficacy of anti-PD1 therapy in cancer treatment. Ascorbic acid (AA, vitamin C) has been previously shown to cause genome-wide demethylation in multiple malignancies by enhancing the activity of the Ten-Eleven Translocation (TET) enzymes. This study shows that AA treatment 1) increases immunogenicity of lymphoma cells; 2) enhances intratumoral infiltration of CD8+ T cells and macrophages; and 3) synergizes with anti-PD1 checkpoint inhibition in a syngeneic lymphoma mouse model via marked activation of cytotoxic cells (cytotoxic T cells and NK cells) and antigen presenting cells. The data provide a compelling rationale for testing combinations of high-dose AA and anti-PD1 agents in patients with aggressive B cell lymphoma and in preclinical models of other malignancies.

Checkpoint inhibitors, particularly antibodies blocking programmed cell death 1 (anti-PD1) and programmed cell death 1 ligand 1 (anti–PD-L1) proteins, have revolutionized cancer treatment and have gained Food and Drug Administration approval for use in multiple malignancies (1). Patients with an objective response to checkpoint inhibitors appear to have a more sustained response compared to conventional chemotherapy, with a favorable adverse effect and quality of life profile. However, results of checkpoint inhibition have not been as promising in some malignancies, such as aggressive non-Hodgkin lymphomas. Identifying agents with the potential of enhancing the sensitivity of tumors to these agents is therefore of utmost importance.

Epigenetic-targeting agents, particularly DNA methyltransferase inhibitors (DNMTIs), are being investigated in combination with anti-PD1 therapy in hematologic malignancies (2). Enhanced endogenous retroviral expression and cancer testis antigen expression induced by demethylation results in increased tumor recognition by immune cells (3–7). We have previously shown that ascorbic acid (AA) causes demethylation and corresponding increase in the hydroxymethylation fraction of both lymphoma and renal cell carcinoma cells by enhancing the activity of the Ten-Eleven Translocation (TET) enzymes (8, 9). AA has also been shown to enhance endogenous retroviral expression in colorectal cancer cells (10). We hypothesized that AA may be an optimal demethylating agent for combination with anti-PD1 therapy as it has also been shown to enhance the function of immune cells such as natural killer (NK) cells, macrophages, and dendritic cells (11, 12). In contrast, effects of DNMTIs on immune cells are inconsistent, with some studies indicating an inhibitory effect (3, 13–16). Importantly, high-dose AA has been shown to be a promising anticancer agent in early-phase clinical trials and is not only well tolerated but appears to decrease the side effects of chemotherapy (17).

In this study, we characterized genome wide high-resolution methylation changes, endogenous retroviral expression, and PD-L1 expression changes in lymphoma cells with high-dose AA treatment and the subsequent effect on sensitivity to cytotoxic T cell-mediated killing. Given that T lymphocytes exhibit an enrichment of 5-hydroxymethylcytosine (5hmC) at gene bodies during differentiation and development (18), we determined the direct effects of AA on CD8+ T cells with regards to global 5hmC changes and cytotoxic function. Finally, we investigated antitumor effects of AA alone and in combination with anti-PD1 therapy in a syngeneic lymphoma mouse model and determined the changes in the tumor immune microenvironment.

## Results

### AA Treatment Leads to Genomewide Demethylation and Enhanced Endogenous Retroviral Expression in Lymphoma Cells but No Change in PD-L1 Expression.

**Fig. 1.**
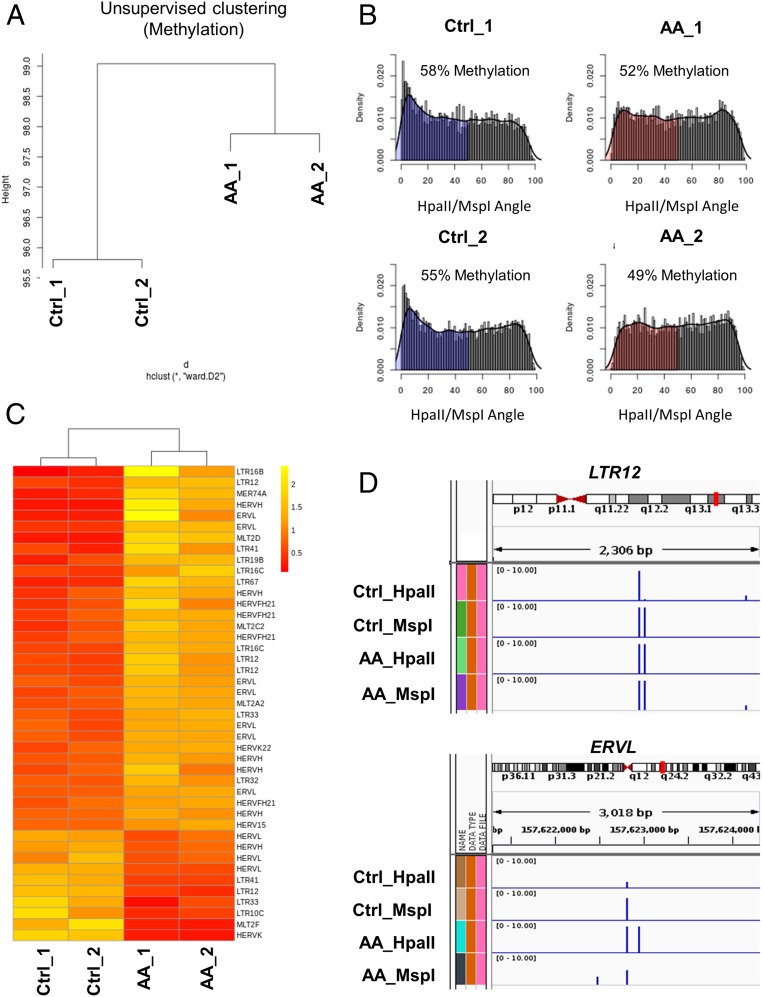
AA treatment leads to genomewide demethylation and enhanced endogenous retroviral expression in lymphoma cells. (*A*) Global methylation analysis was conducted using the HELP assay on OCI-Ly1 cells treated with control (Ctrl) or 1 mM AA for 6 h; cells harvested 24 h after treatment (*n* = 2 replicates per group). Genomewide unsupervised clustering was performed on the resulting cytosine methylation data. Ward clustering shows global methylation changes are induced by AA treatment. The 2 AA-treated samples clustered separate from the 2 Ctrl-treated samples, with consistency between the 2 samples of each treatment. (*B*) Genomewide plot of HELP cytosine methylation (based on HpaII/MspI angle) in OCI-Ly1 Ctrl and AA samples. A reduction in the loci with cytosine methylation (shaded regions; HpaII/MSPI angle <50) was observed with AA treatment, reflecting a significant decrease in genomewide methylation levels with AA treatment. (reduction in methylation from 58% in Ctrl_1–52% in AA_1; reduction in methylation from 55% in Ctrl_2–49% in AA_2; *t* test, *P* = 0.05; gray represents hypomethylated loci and blue and red represent methylated loci). (*C*) HERV expression was assessed by RNA sequencing in Ctrl and AA treated OCI-Ly1 cells. A heat map of differentially expressed HERVs between Ctrl and AA groups showed that AA treatment enhanced expression of the majority of differentially expressed HERVs (∼70%). The cutoff for differential expression was defined as *P* < 0.01. (*D*) HELP methylation loci of LTR12 and ERVL HERVs (representative) revealing an increase in HpaII counts, that is, demethylation, with AA treatment. (Global methylation and ERVs correlation are depicted in *SI Appendix*, Fig. S1.)

We previously demonstrated that AA treatment resulted in TET-mediated demethylation and increased hydroxymethylation in lymphoma cells, using both the enzyme-linked immunosorbent assay–based TET activity assay and liquid chromatography electrospray ionization tandem mass spectrometry (MS/MS) (8). Diffuse large B cell lymphomas underexpress *TET2* relative to control lymphocytes (*SI Appendix*, Fig. S1*A*), and it has recently been shown that TET2 deficiency promotes lymphomagenesis (19), underscoring the rationale to enhance activity of this tumor-suppressor gene. Here, we further characterized the demethylation effect with high-resolution methylation analysis using the HELP (Hpa II tiny fragment enrichment by ligation-mediated PCR) assay that relies on differential restriction digestion of methylated CpGs followed by high-throughput sequencing analysis. Unsupervised clustering demonstrated that AA treatment led to changes in cytosine methylation patterns with epigenetic dissimilarity between control and AA-treated lymphoma cells (Fig. 1*A*). Specifically, AA treatment led to global loss of cytosine methylation (Fig. 1*B*). We next assessed the expression of human endogenous retroviruses (HERVs) by RNA sequencing. HERVs have been shown to be up-regulated by hypomethylating agents (10). HERVs increase immune recognition of tumor cells, trigger an interferon response by induction of the viral defense pathway and enhance checkpoint blockade antitumor activity (20, 21). Consistent with global loss of methylation, the majority (70%) of differentially expressed HERVs were increased in lymphoma cells following AA treatment (Fig. 1*C*). Global methylation analysis of HERVs up-regulated with AA treatment revealed that ∼60% of the loci were demethylated upon AA treatment (*SI Appendix*, Fig. S1*C*). The loci of ERVL and LTR12 HERVs (representative) revealed baseline DNA hypermethylation that was subsequently demethylated upon AA treatment (Fig. 1*D*). Furthermore, AA up-regulated HERVs were associated with a higher proportion of demethylated loci when compared to the down-regulated HERVSs (27% difference between AA up-regulated and down-regulated HERV-associated methylation) (*SI Appendix*, Fig. S1*D*).

Expression of PD-L1 (encoded by *CD274*) has been shown to be regulated, in part, by DNA methylation and as such is up-regulated in tumor cells by DNMTIs (22). We therefore hypothesized that AA treatment may lead to similar increase in PD-L1 expression via TET-mediated demethylation of the *CD274* locus. However, there was no increase in PD-L1 expression with AA treatment in any of the 4 DLBCL cell lines tested (OCI-Ly1, OCI-Ly5, OCI-Ly7, and SU-DHL6) as measured by RT-PCR (*SI Appendix*, Fig. S1*B*). Furthermore, there was no change in methylation at the *CD274* locus with AA treatment of the OCI-Ly1 cell line.

### AA Pretreatment of Lymphoma Cells Leads to Increased Sensitivity to CD8+ T Cell Cytotoxicity.

**Fig. 2.**
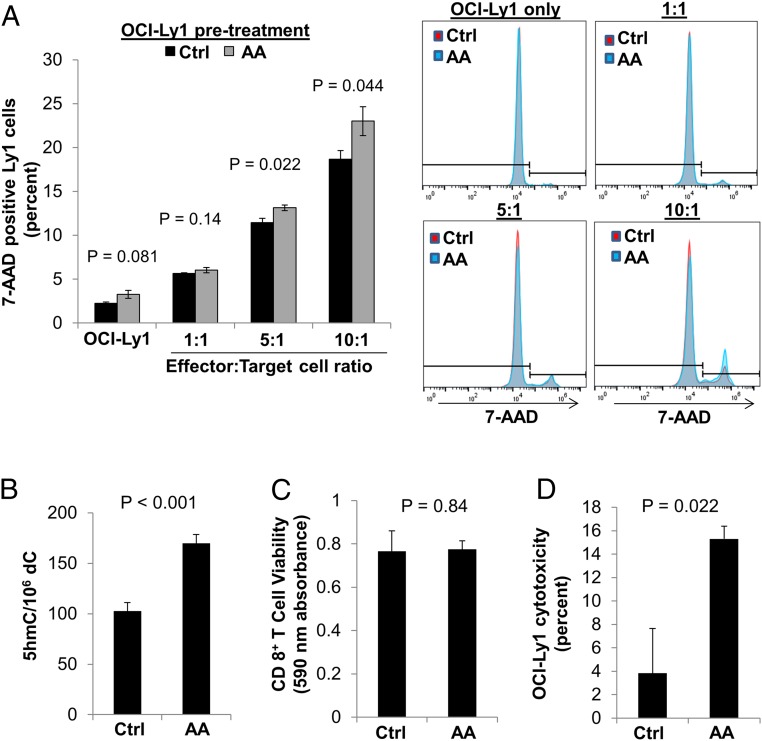
Pretreatment of both lymphoma cells or CD8+ T cells with AA enhances T cell-mediated lymphoma cell cytotoxicity. (*A*) Pretreatment of OCI-Ly1 B cell lymphoma cells with AA did not significantly affect B cell viability (*P* = 0.081) but increased immunogenicity in a T cell cytotoxicity assay (5:1 T:B cell ratio, *P* = 0.022; 10:1 ratio, *P* = 0.044). OCI-Ly1 cells were pretreated with control (Ctrl) or AA for 6 h. OCI-Ly1 cells (target cells) were then suspended in fresh medium with specified ratios of CD8+ T cells (effector cells) and incubated for 48 h. OCI-Ly1 cytotoxicity was measured by 7-AAD positive staining in OCI-LY1 cells. Each condition was performed in triplicate and representative flow cytometry is shown. (*B*) AA significantly increased 5hmC in CD8+ T cells compared to Ctrl (*P* < 0.001, paired *t* test) as measured by MS. CD8+ T cells isolated from 3 normal donors were treated with Ctrl or AA for 6 h and cells were harvested at 24 h after treatment. (*C*) Treatment of CD8+ T cells with AA for 6 h had no impact on T cell viability 24 h after treatment (*P* = 0.84) as measured by Alamar Blue cell viability assay. (*D*) Pretreatment of CD8+ T cells with AA increased B cell lymphoma cytotoxicity (*P* = 0.022) as measured by LDH cytotoxicity assay. CD8+ T cells were pretreated with Ctrl or AA for 6 h, then CD8+ T cells were combined with OCI-Ly1 cells in a 1:1 ratio for 24 h. Data are expressed as means ± SEM.

Given the findings of AA-induced demethylation and increased HERV expression in lymphoma cells, we sought to determine whether AA-pretreated lymphoma cells were more sensitive to cytotoxic T cell-mediated killing. To test this, we pretreated OCI-Ly1 lymphoma (target) cells with 0 or 1 mM AA and combined them with CD8^+^ T (effector) cells derived from healthy donors in various ratios of effector:target cells. Indeed, we found that pretreatment of lymphoma cells with high-dose AA significantly increased their immunogenicity as evidenced by increased percent killing of lymphoma cells by 15% and 21% of control by CD8+ T cells when combined at 5:1 and 10:1 effector:target cell ratios, respectively (*t* test, *P* < 0.05; Fig. 2*A*).

### AA Treatment of CD8+ T Lymphocytes Leads to Increase in Hydroxymethylcytosine Fraction (5hmC/C) and Enhancement of Its Cytotoxic Activity Against Lymphoma Cells.

T lymphocytes have been previously shown to have an enrichment of 5hmC at gene bodies, with dynamic changes during differentiation and development. Hence, we hypothesized that AA treatment of CD8^+^ T cells would lead to an increase in the 5hmC fraction and that it may be associated with enhanced cytotoxic activity.

As hypothesized, isolated CD8+ T cells from 3 healthy individuals revealed a significant global increase in the 5hmC fraction with AA treatment, measured by MS (103 ± 5 vs. 170 ± 5hmC/10^6^ C, paired *t* test, *P* < 0.001; Fig. 2*B*). Viability of CD8+ T cells was not altered by AA treatment (*P* = 0.84; Fig. 2*C*). To assess whether CD8+ T cell function was altered by AA, we performed coculture cytotoxicity experiments with OCI-Ly1 lymphoma cells. AA pretreatment of healthy donor-derived CD8+ T cells led to a 3.8-fold increase in their cytotoxic activity against lymphoma cells, as measured with the LDH (lactate dehydrogenase) cytotoxicity assay (*P* = 0.022; Fig. 2*D*). We validated this finding using a flow cytometry-based cytotoxicity assay in which AA pretreatment of CD8+ T cells increased 7-AAD uptake of OCI-Ly1 cells (*SI Appendix*, Fig. S2).

### High-Dose AA Treatment Synergizes with Anti-PD1 Checkpoint Inhibition in a Syngeneic Lymphoma Mouse Model, Resulting in Significant Tumor Proliferation Inhibition.

**Fig. 3.**
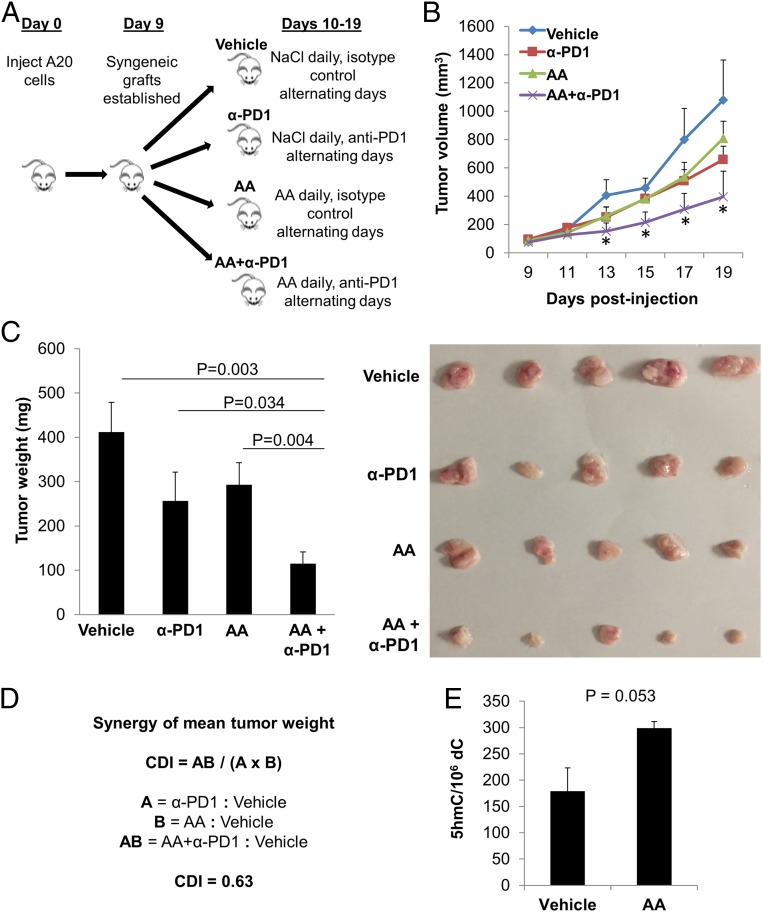
High-dose AA treatment synergizes with anti-PD1 checkpoint inhibition in a syngeneic lymphoma mouse model. (*A*) Forty BALB/c mice received an s.c. right flank injection of 5 × 10^6^ A20 tumor cells in PBS and were randomly assigned to 4 groups of 10 mice: vehicle, anti-PD1 (α-PD1), AA, or AA+α-PD1. On day 9, palpable tumors were observed in 7 to 9 mice in each group (vehicle, *n* = 7; α-PD1, *n* = 9; AA, *n* = 9; AA+α-PD1, *n* = 8). Daily treatment was administered from day 10 until the tumor size endpoint was met. (*B*) Tumor volume (cubic millimeters) was monitored every 2 d over the duration of the study by caliper and calculated as (Width^2^ × Length)/2, where W and L represent the shorter and longer diameters (millimeters), respectively. See all individual tumor growth curves in *SI Appendix*, Fig. S3. Given the highly aggressive nature of the A20 lymphoma mouse model, humane endpoint was reached in one mouse in the vehicle group on day 19 (after only 9 d of treatment). To facilitate comparison between the treatment groups, all mice in the 4 groups were killed on day 19 and tumors excised and weighed. The growth curve of the AA+α-PD1 group was significantly divergent from that of the vehicle group starting on day 13 (day 4 of treatment) and continuing through the end of the study (*t* test *P* values between AA+α-PD1 and vehicle groups on days 13, 15, 17, and 19 were 0.042, 0.016, 0.029, and 0.028 respectively; asterisk represents <0.05). On the other hand, the growth curves of neither single-agent α-PD1 nor single-agent AA were significantly divergent (statistically) compared to that of the vehicle group at any point, but both demonstrated a trend toward proliferation inhibition compared to the vehicle group. Single-agent α-PD1 vs. vehicle approached statistical significance with a *P* value of 0.069 at the end of the study on day 19. (*C*) Tumor weight was significantly lower in the combined treatment group, AA+α-PD1, compared to vehicle (*P* = 0.003), α-PD1 (*P* = 0.034), and AA (*P* = 0.004) groups (ANOVA, *P* = 0.025). Included in *C* is a representative picture of 5 tumors in each group, which were further studied for intratumoral epigenomic and immune microenvironment analyses. (*D*) CDI was calculated to test for synergy between AA and α-PD1 using mean tumor weight measurements. The CDI value of 0.63 indicates synergy (defined as CDI < 1, with CDI < 0.7 indicating a significantly synergistic effect). (*E*) Global 5hmC was elevated in tumors of mice treated with AA compared to vehicle (*P* = 0.053) as measured by MS. Data are expressed as means ± SEM.

In vitro experiments, however sophisticated, cannot replicate the complex interactions between tumor cells and the comprehensive immune microenvironment. As such, we employed a preclinical in vivo model to further investigate potential mechanisms of cooperation between high-dose AA and anti-PD1 treatment. Using the A20 lymphoma syngeneic mouse model, we treated tumor-bearing mice with vehicle, anti-PD1, high-dose AA, or the combination of high-dose AA and anti-PD1 (AA+anti-PD1) (Fig. 3*A*). Daily treatment was administered from day 10 until the tumor size endpoint was met. Tumor volume was measured by caliper (cubic millimeters) once tumors were palpable (day 9) and continuing through the end of the study (Fig. 3*B*; also see *SI Appendix*, Fig. S3). Given the highly aggressive nature of the A20 lymphoma mouse model, humane endpoint was reached in one mouse in the vehicle group on day 19 (after only 9 d of treatment). To facilitate comparison between the treatment groups, all mice in the 4 groups were killed on day 19 and tumors were excised and weighed.

The growth curve of the AA+α-PD1 group was significantly divergent from that of the vehicle group starting on day 13 (day 4 of treatment) and continuing through the end of the study (*t* test *P* values between AA+α-PD1 and vehicle groups on days 13, 15, 17, and 19 were 0.042, 0.016, 0.029, and 0.028 respectively; *P < 0.05). On the other hand, the growth curves of neither single-agent α-PD1 nor single-agent AA were significantly divergent (statistically) compared to that of the vehicle group at any point, but both demonstrated a trend toward proliferation inhibition compared to the vehicle group. Single-agent α-PD1 vs. vehicle approached statistical significance with a *P* value of 0.069 at the end of the study on day 19 (Fig. 3*B*).

Tumor weight was significantly lower in the combined treatment group, AA+α-PD1, compared to vehicle (*P* = 0.003), α-PD1 (*P* = 0.034), and AA (*P* = 0.004) groups (ANOVA, *P* = 0.025). Because the observed effect of combined AA and anti-PD1 therapies was greater than the expected additive effect (as seen in Fig. 3*C*) we applied the coefficient of drug interaction (CDI) formula (23–27) to calculate synergy using mean tumor weight measurements. The CDI between high-dose AA and anti-PD1 using the mean tumor weight measurements was 0.63, indicating a significantly synergistic effect (CDI < 0.7).

We next investigated whether in vivo AA treatment altered intratumoral DNA methylation as we had shown in vitro for lymphoma cells (Fig. 1). Using MS we showed that AA treatment resulted in a 1.67-fold increase in the intratumoral 5hmC fraction compared to vehicle (*t* test, *P* = 0.05; Fig. 3*E*), consistent with the demethylation observed in vitro, and also consistent with the parenteral AA-induced increase in intratumoral 5hmC we have previously reported in a kidney cancer xenograft model (9).

### High-Dose AA and Anti-PD1 Treatment Combination Leads to Increase in Tumor CD8+ T Cell and Macrophage Infiltration, Enhanced Granzyme B Production by Cytotoxic Cells, and Enhanced Interleukin 12 Production by Antigen-Presenting Cells.

To characterize changes in the tumor microenvironment associated with the synergistic antitumor activity of AA and anti-PD1, we performed immunofluorescence for markers of immune cell infiltration and function and quantified the staining with artificial intelligence (AI) technology.

**Fig. 4.**
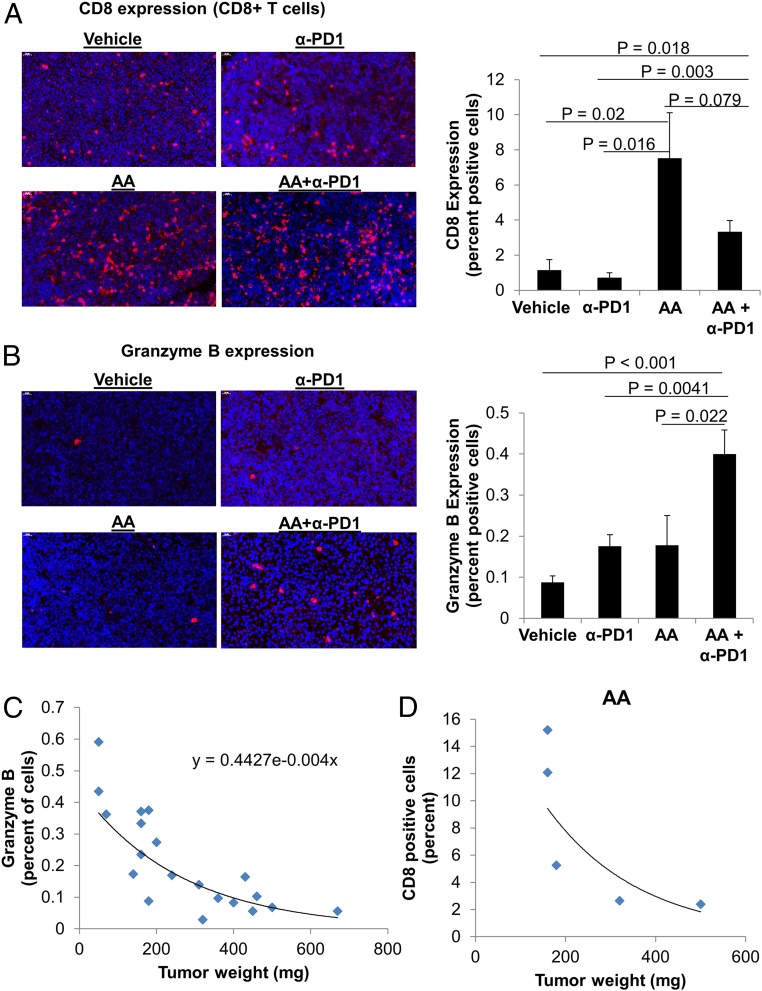
High-dose AA and anti-PD1 treatment combination leads to increase in tumor CD8 + T cell infiltration and enhanced granzyme B production. (*A*) The infiltration of CD8+ T cells was elevated in tumors of mice treated with AA. While AA and AA+α-PD1 were not statistically different (*P* = 0.079), tumors from mice treated with AA+α-PD1 had significantly greater percent CD8+ T cells compared to vehicle (*P* = 0.018) and α-PD1 (*P* = 0.003) (ANOVA, *P* = 0.012). CD8 staining was performed on mouse tumors by immunofluorescence, and images were captured at 50× magnification. See also *SI Appendix*, Fig. S4. (*B*) The percent of cells expressing granzyme B was significantly higher in AA+α-PD1 relative to vehicle (*P* < 0.001), α- PD1 (*P* = 0.004), and AA (*P* = 0.022) groups (ANOVA, *P* = 0.003). Data are expressed as means ± SEM. Granzyme B immunofluorescence images were captured at 50× magnification. See also *SI Appendix*, Fig. S5. (*C*) Across all treatment groups, tumor granzyme B expression was negatively correlated with tumor weight, with an exponential increase in weight with decreasing granzyme B expression. (*D*) Within the AA group, smaller tumors were associated with higher percent of intratumoral CD8+ T cells. Data are expressed as means ± SEM of 5 representative mouse tumors per treatment group; all tumors were harvested on day 19 (day 9 after treatment initiation). Immunofluorescence staining was quantified by artificial intelligence technology.

Immunofluorescence for CD8 revealed increased infiltration of CD8+ T cells in the groups that received AA, irrespective of anti-PD1 treatment (Fig. 4*A*). CD8^+^ T cell infiltration was significantly higher for the AA group (7.5 ± 2.6% percent positive cells; mean ± SEM) compared with vehicle (1.2 ± 0.6%, *t* test, *P* = 0.02) or anti-PD1 groups (0.7 ± 0.3%, *t* test, *P* = 0.016). The CD8+ T cell infiltration with single-agent AA treatment was not statistically different from the AA+anti-PD1 group (3.34 ± 0.64%, *P* = 0.079). CD8+ T cell infiltration in the AA+anti-PD1 group was significantly higher than vehicle (*t* test, *P* = 0.018) and anti-PD1 treatment (*t* test, *P* = 0.003) (Fig. 4*A*; also see *SI Appendix*, Fig. S4).

Cytotoxic function of the tumor microenvironment was assessed by granzyme B staining. Granzyme B production within the tumor microenvironment of the AA+anti-PD1 group (0.4 ± 0.06%) was significantly higher than AA alone (0.18 ± 0.07%, *t* test, *P* = 0.02), anti-PD1 alone (0.18 ± 0.03%, *t* test, *P* = 0.004), and vehicle (0.09 ± 0.015%, *t* test, *P* < 0.001) (Fig. 4*B*; also see *SI Appendix*, Fig. S5). Granzyme B positivity within the tumor microenvironment correlated inversely with tumor weight, with an exponential increase in tumor weight with decreasing granzyme B expression (*R*^2^ = 0.75) (Fig. 4*C*). Within the AA treatment group, the larger tumors had lesser CD8+ T cell infiltration (Fig. 4*D*).

**Fig. 5.**
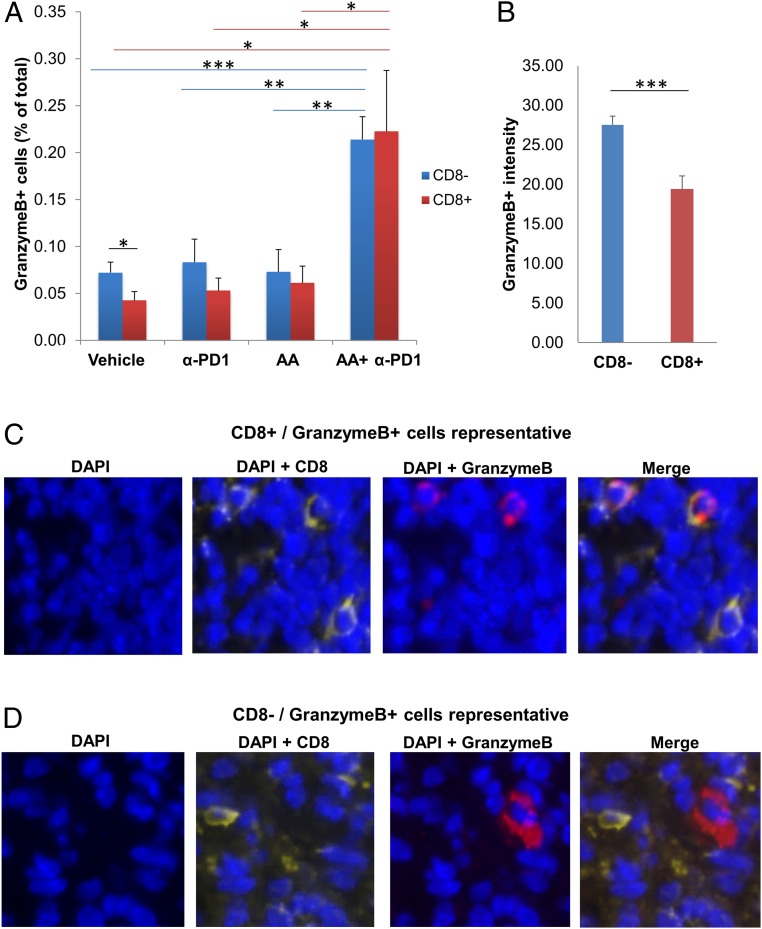
High-dose AA and anti-PD1 treatment combination activates cytotoxic CD8+T cells and cytotoxic NK cells/CD8− T cells. The contribution of NK cells/CD8− T cells toward net granzyme B production is more than CD8+ T cells. (*A*) Mouse tumors were costained with CD8 and granzyme B. The percent CD8−/granzyme B+ percent positive cells (representing NK cells/CD8− T cells with granzyme B) was not significantly different from the CD8+/granzyme B+ percent positive cells (representing CD8+ T cells with granzyme B) within each of 3 treatment groups: AA, anti-PD1, AA+anti-PD1. In the vehicle group, CD8−/ granzyme B+ percent positive cells was higher than CD8+/granzyme B+ percent positive cells (*P* = 0.038). The CD8+/ granzyme B+ cell count in the AA+α-PD1 group was significantly higher than in vehicle (*P* = 0.012), single-agent AA (*P* = 0.021), and single-agent α-PD1 (*P* = 0.016). Similarly, CD8−/granzyme B+ cell count in the AA+α-PD1 group was significantly higher than in vehicle (*P* < 0.001), single-agent AA (*P* = 0.002), and single-agent anti-PD1 (*P* = 0.003). Immunofluorescence images were captured at 50× magnification. *P < 0.05; **P < 0.005; ***P < 0.001. (*B*) Intensity of granzyme B staining within the CD8− cells across all treatment groups was significantly higher than within the CD8+ cells (*P* < 0.001). Data expressed as means ± SEM of 5 representative mouse tumors per treatment group; all tumors were harvested on day 19 (day 9 after treatment initiation). ***P < 0.001. (*C* and *D*) Magnified (80×) images of dual CD8 and granzyme B costain showing representative pictures of individual CD8+/granzyme B+ cells (*C*) and CD8−/granzyme B+ cells (*D*). The amount of granzyme B within CD8− cells appears to be much higher than within CD8+ cells, explaining the difference in intensity calculated in B. Immunofluorescence staining was quantified by artificial intelligence technology (*A* and *B*).

Apart from CD8+ T cells, granzyme B has been shown to be produced by NK cells (28, 29) and to a small extent by CD8− T cells (29). We therefore aimed to determine the relative contribution of CD8+ T cells and NK cells/CD8− T cells toward granzyme B production, and the effect of the combination treatment in activation of CD8+ T cells and NK cells/CD8− T cells. Given the lack of a specific antibody for NK cells in BALB/c mice, we used CD8/granzyme B costaining to determine the granzyme B contribution from NK cells/CD8− T cells (CD8−/granzyme B+) and CD8+ T cells (CD8+/granzyme B+). We found that the CD8−/granzyme B+ percent positive cells (representing NK cells or CD8− T cells with granzyme B) was not significantly different from the CD8+/granzyme B+ percent positive cells (representing CD8+ T cells with granzyme B) within each of 3 treatment groups: AA, anti-PD1, and AA+anti-PD1 (Fig. 5*A*). In the vehicle group, CD8−/granzyme B+ percent positive cells (0.07 ± 0.01%) was higher than CD8+/granzyme B+ percent positive cells (0.04 ± 0.01%, *t* test, *P* = 0.038) (Fig. 5*A*). The intensity of granzyme B staining within the CD8− cells across all treatment groups (27.53 ± 1.09) was significantly higher than within the CD8+ cells (19.37 ± 1.7, *t* test, *P* < 0.001) (Fig. 5*B*). Magnified (80×) images of dual CD8 and granzyme B costain showing representative pictures of individual CD8+/granzyme B+ cells (Fig. 5*C*) and CD8−/granzyme B+ cells (Fig. 5*D*) reveal that the amount of granzyme B within CD8− cells appears to be much higher than within CD8+ cells, explaining the difference in intensity calculated in Fig. 5*B*. Put together, the contribution of NK cells/CD8− T cells toward the cumulative cytotoxic function across all treatment groups was more than CD8+ T cells.

The CD8+/granzyme B+ cell count in the AA+anti-PD1 group (0.22 ± 0.06%) was significantly higher than in vehicle (0.04 ± 0.01%, *t* test, *P* = 0.012), single-agent AA (0.06 ± 0.02%, *t* test, *P* = 0.021), and single-agent anti-PD1 (0.05 ± 0.01%, *t* test, *P* = 0.016) (Fig. 5*A*). Similarly, CD8−/granzyme B+ cell count in the AA+anti-PD1 group (0.21 ± 0.02%) was significantly higher than in vehicle (0.07 ± 0.01%, *t* test, *P* < 0.001), single-agent AA (0.07 ± 0.02%, *t* test, *P* = 0.002), and single-agent anti-PD1 (0.08 ± 0.02%, *t* test, *P* = 0.003) (Fig. 5*A*). Taken together, the combination treatment resulted in marked activation of cytotoxic function of both cytotoxic T cells and NK cells. [Of note, the total (CD8+ and CD8−) granzyme B percent positive cells detected as a costain for each treatment group was about the same as that detected by single stain, serving as an additional quality check for the immunofluorescence staining and AI quantitation.]

**Fig. 6.**
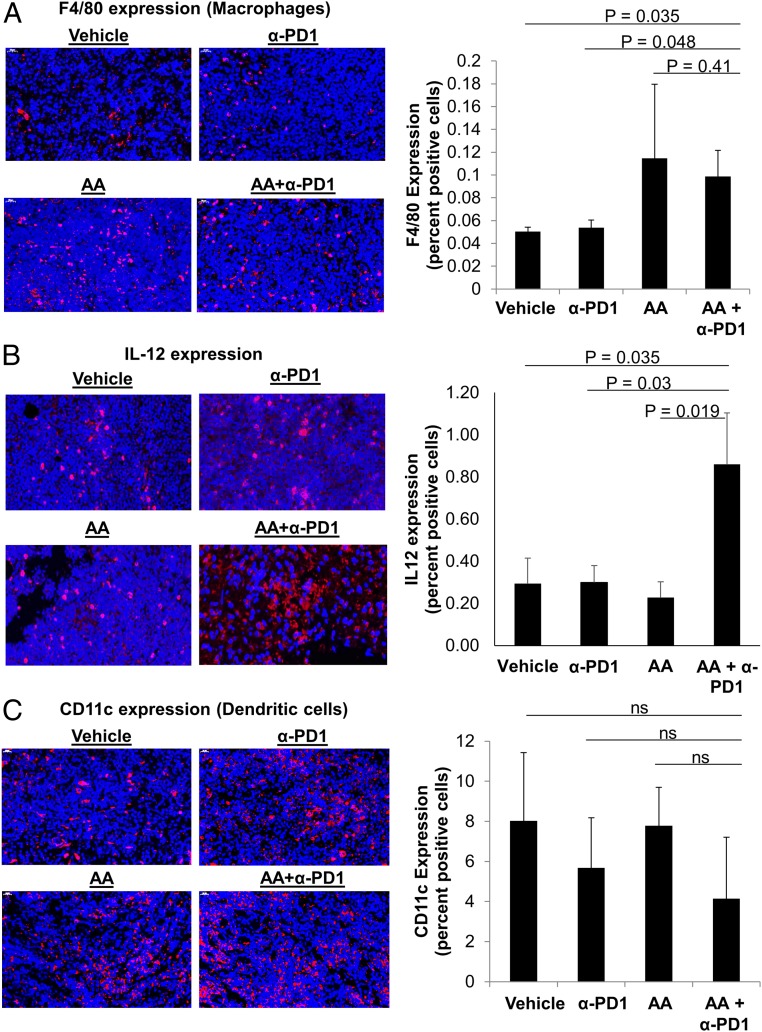
High-dose AA and anti-PD1 treatment combination leads to increase in tumor macrophage infiltration and IL-12 production but does not change dendritic cell infiltration. (*A*) The mouse macrophage marker, F4/80, was increased in mice receiving AA either as a single agent or combination. The percent of intratumoral macrophages was higher in AA+α-PD1 compared to vehicle (*P* = 0.035) and α-PD1 alone (*P* = 0.048). See also *SI Appendix*, Fig. S6. (*B*) IL-12 (a cytokine produced by activated APCs and in turn activates cytotoxic T cells and NK cells) was significantly increased in the AA+ α-PD1 group compared to α-PD1 alone (*P* = 0.03), AA alone (*P* = 0.019), and vehicle (*P* = 0.035). (*C*) CD11c (dendritic cell) expression was not significantly different between treatment groups (ANOVA, *P* > 0.1). See also *SI Appendix*, Fig. S7. Data expressed as means ± SEM of 5 representative mouse tumors per treatment group; all tumors were harvested on day 19 (day 9 after treatment initiation). Immunofluorescence images were captured at 50× magnification, as indicated. ns, not significant. Immunofluorescence staining was quantified by artificial intelligence technology.

Macrophage infiltration was determined by the mouse macrophage marker F4/80. Similar to the pattern observed for CD8, macrophage infiltration with AA+anti-PD1 treatment (0.1 ± 0.02%) was significantly higher than vehicle treatment (0.05 ± 0.003%, *t* test, *P* = 0.035) or anti-PD1 alone (0.05 ± 0.006%, *t* test, *P* = 0.048) but not statistically different from AA alone (0.11 ± 0.06%, *t* test, *P* = 0.41) (Fig. 6*A*; also see *SI Appendix*, Fig. S6). We assessed dendritic cell infiltration with CD11c. CD11c expression did not differ across different treatments (Fig. 6*C*; also see *SI Appendix*, Fig. S7).

Interleukin 12 (IL-12) staining was done to assess the activation and function of intratumoral antigen-presenting cells (APCs) (macrophages and dendritic cells). IL-12 is produced by APCs and activates both CD8+ T cells (30) and NK cells (31, 32). Similar to the pattern observed for granzyme B, IL-12 production within the tumor microenvironment of the AA+anti-PD1 group (0.86% ± 0.24) was significantly higher than anti-PD1 alone (0.3% ± 0.08, *t* test, *P* = 0.03), AA alone (0.23% ± 0.07, *t* test, *P* = 0.019), and vehicle (0.29% ± 0.12, *t* test, *P* = 0.035) (Fig. 6*B*).

Consistent with our in vitro experiments, PD-L1 expression was not induced by AA and did not differ across different treatments (*SI Appendix*, Fig. S8).

## Discussion

Clinical trials with checkpoint inhibitors, particularly anti-PD1/PD-L1, have yielded impressive response rates in several tumor types. As such, these agents are currently approved for the treatment of several malignancies. Efforts to further improve the efficacy by combining these drugs with potentiating agents/modalities are ongoing—both in malignancies in which they have been approved for clinical use and in malignancies where single-agent checkpoint inhibition has not demonstrated significant benefit. These efforts are directed at overcoming mechanisms limiting responsiveness to checkpoint inhibition. These mechanisms include 1) poor immune recognition of tumors, especially those with low tumor mutational burden and neoantigen expression, resulting in lower tumor infiltrating lymphocytes and macrophages; 2) inhibitory effect of tumor cells on immune cells (both within and outside the tumor microenvironment) via cytokines and aberrant metabolic intermediates; 3) presence of an abnormally high percentage of inhibitory regulatory T cells and myeloid-derived suppressor cells (MDSCs); and 4) inhibition of cytotoxic T lymphocytes with mechanisms other than PD1/PD-L1 interaction, including expression of other inhibitory molecules, epigenetic reprograming, and lack of stimulation by activated dendritic cells/macrophages.

Our results indicate that high-dose AA treatment overcomes several of the above checkpoint inhibition resistance mechanisms. First, by enhancing the expression of HERVs via demethylation, it appears to enhance immune recognition of tumor cells, as evidenced by an increase in macrophage and cytotoxic T cell infiltration within the tumor microenvironment. The intratumoral infiltration of CD8+ T cells was significantly higher with single-agent high-dose AA treatment compared with single-agent vehicle or anti-PD1 treatment. Second, AA treatment of cytotoxic T cells resulted in an increase in its cytotoxic activity in vitro, associated with an increase in its hydroxymethylcytosine fraction (as seen in activated lymphocytes). These findings are additionally supported by the enhanced granzyme B production by cytotoxic T cells in the tumor microenvironment with single-agent AA compared with vehicle treatment. Furthermore, the granzyme B production with combined high-dose AA and anti-PD1 treatment was significantly higher than either agent alone. Third, previously reported in vitro studies on the effects of AA treatment on other immune cells (dendritic cells, macrophages, and NK cells) have suggested an increase in the activity of these immune cells (11, 12). Indeed, our data reveal that high-dose AA when added to anti-PD1 activates not only cytotoxic T cells but also NK cells and APCs (dendritic cells and macrophages).

### Synergistic Mechanisms Resulting in Synergistic Antitumor Effect.

**Fig. 7.**
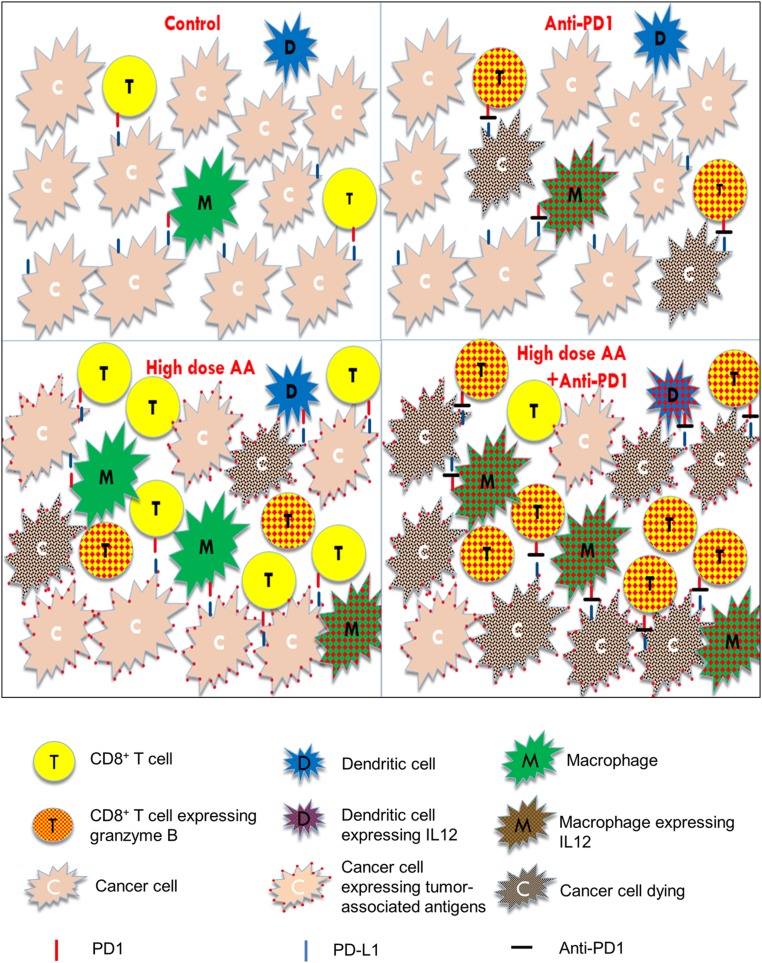
Graphical summary of the synergistic effects of high-dose AA and anti-PD1. Single-agent anti-PD1**:** blocks the inhibitory PD-1/PD-L1 axis, thereby activating the sparse infiltrated cytotoxic T lymphocytes, NK cells (not depicted), and macrophages. Single-agent high-dose AA**:** enhances tumor immune recognition and increases macrophage and cytotoxic T cell infiltration into the tumor. However, despite the increased immune cell numbers, the inhibitory effect of PD-1/PDL-1 axis on cytotoxic cells and macrophages cannot be abrogated by AA. Anti-PD1+high-dose AA**:** The combination treatment results in both increased immune infiltration and enhanced activation of APCs and cytotoxic cells, resulting in marked tumor shrinkage. Red dots within the T cells represent granzyme B. Red dots within the macrophages and dendritic cells represent IL-12, which activates the cytotoxic cells (cytotoxic T cells and NK cells). Red dots on the surface of cancer cells represent increased HERV expression with AA treatment (or other potential immune-recognition enhancing antigenic epitopes). Black dots within the cancer cells represent dying cells. Red mark on T cells and macrophages represents PD1. Blue mark on cancer cells represents PDL1. Black mark between the red and blue marks represents anti-PD1 antibody.

High-dose AA treatment enhances tumor immune recognition, resulting in increased intratumoral infiltration of macrophages and cytotoxic T cells, findings not observed with anti-PD1 treatment alone. Anti-PD1 blocks the inhibitory effect of PD-1/PD-L1 axis on APCs, CD8+ T cells, and NK cells and is more potent at directly activating these cells than high-dose AA. The combination treatment results in markedly higher IL-12 production by APCs and granzyme B production by cytotoxic cells (cytotoxic T cells and NK cells) than either agent alone. Taken together, these results suggest synergistic mechanisms of antitumor action with high-dose AA and anti-PD1 treatment (see Fig. 7 for a graphical summary).

The growth curve of the AA+α-PD1 group was significantly divergent from that of the vehicle group starting on day 4 of treatment (day 13) and continuing through the end of the study (day 19) (Fig. 3*B*). On the other hand, the growth curves of neither single-agent α-PD1 nor single-agent AA were significantly divergent (statistically) compared to that of the vehicle group, but both demonstrated a trend toward proliferation inhibition. Furthermore, we observed markedly smaller tumors with AA+ anti-PD1 combination therapy compared to either agent alone (Fig. 3*C*). Although synergy calculations in mouse models are not common practice, the observed effect of the combination treatment appeared greater than the expected additive effect from either agent alone, which prompted us to perform synergy calculations based on the mean tumor weight. Also, the mechanisms detailed above suggested a synergistic effect. We found that the combination treatment indeed had a synergistic antitumor effect in this lymphoma mouse model (CDI = 0.63). This synergistic effect was in the context of a highly aggressive syngeneic lymphoma model (A20) in which humane endpoint was reached only 9 d after initiation of treatment. Given the aggressiveness of the A20 model, the differences and synergy observed are particularly meaningful. Importantly, despite the aggressiveness of the A20 model, we waited until tumors were palpable before starting treatment.

Granzyme B expression in cytotoxic cells is dynamic, and expression measured by staining only captures the quantity of expression at that particular time point. Our finding that the magnitude of granzyme B expression within the cytotoxic cells increased by 3 to 4 times with the combination (AA+ anti-PD1) compared with single-agent AA or anti-PD1 is biologically (as well as statistically) very significant. Consistent with the report of granzyme B’s being a useful predictive imaging biomarker for immunotherapy (33), our data showed that granzyme B expression negatively correlated with tumor weight, with an exponential increase in weight with decreasing granzyme B expression (Fig. 4*C*).

Interestingly, anti-CTLA4 was recently tested in combination with anti-PD1 in a syngeneic mouse model (29). The anti-CTLA4/anti-PD1 combination treatment did not result in either an additive or synergistic antitumor effect (29). The anti-CTLA4/anti-PD1 combination neither increased intratumoral CD8+ T cell infiltration nor granzyme B production within CD8+ T cells or NK cells, when compared to single-agent anti-PD1 (29). Although it was a different syngeneic mouse model from the one used in our study, the data provide further context to the significance of our findings with the AA+anti-PD1 combination.

### More than Just CD8+ T Cell Activation.

NK cells are known to be inhibited by the PD-1/PD-L1 axis and contribute to effects of PD-1/PD-L1 blockade along with cytotoxic T cells (28, 29). Our data indicate that NK cells and CD8− T cells combined contribute to the overall granzyme B production more than CD8+ T cells across all 4 treatment groups. The intensity of granzyme B staining was significantly higher in NK cells/CD8− T cells (CD8−/granzyme B+ cells) than in CD8+ T cells (CD8+/granzyme B+ cells) (Fig. 5*B*). The percent positive CD8−/granzyme B+ cells was not significantly different from the percent positive CD8+/granzyme B+ cells within each of 3 treatment groups,AA, anti-PD1, and AA+anti-PD1 (Fig. 5*A*), but within the vehicle group CD8−/granzyme B+ percent positive cells was higher than CD8+/granzyme B+ percent positive cells. CD8−/granzyme B+ cells were seen to harbor more granzyme B in their cytoplasm than CD8+/granzyme B+ cells (representative, Fig. 5 *C* and *D*), explaining the difference in intensity of granzyme B staining. Put together, these data suggest that the role of NK cells/CD8− T cells (combined) in anticancer cytotoxicity is at least as significant as CD8+ T cells, if not more.

The AA+anti-PD1 group was found to have significantly higher CD8−/granzyme B+ percent positive cells than other groups and also significantly higher CD8+/granzyme B+ percent positive cells than other groups, indicating that the combination treatment markedly activated both cytotoxic T cells and NK cells.

Similar to NK cells, the PD-1/PD-L1 axis has also been shown to inhibit tumor-associated macrophages (34) and dendritic cells (23). In this lymphoma mouse model, high-dose AA in combination with anti-PD1 not only enhanced intratumoral macrophage infiltration but also increased production of intratumoral IL-12, a cytokine produced by activated APCs, which in turn activates cytotoxic T cells (30) and NK cells (31, 32). Cross-talk between T cells and dendritic cells involving IL-12 has been recently shown to be crucial for successful anti-PD1 therapy (35).

We acknowledge the possible contribution of other mechanisms of action in the antitumor response to AA and its synergy with anti-PD1 that were not measured in this study. For example, it is possible that global demethylation altered expression of other tumor-associated antigens such as mutated peptides or cancer testis antigens, as previously reported for other demethylating agents (36–40). In addition, it is possible that the changes observed in HERV expression could also be mediated by nonmethylation mechanisms. Of note, AA has been shown to have anticancer activity in immunocompromised mouse models, indicating the additional importance of non-immune-mediated mechanisms of action such as oxidative stress, epigenetic reprogramming of cancer cells and the varied cofactor functions of AA (9, 17, 41–45). Given these additional potential contributing mechanisms, we felt that conventional DNMT inhibitors would not serve as appropriate enough controls to justify their inclusion in the in vivo study (and killing 20 more mice), particularly given that the combination of DNMT inhibitors and checkpoint blockade is already being investigated in the clinical setting.

Future work is warranted to further characterize the changes in the immune microenvironment subpopulations and molecular mechanisms of AA-induced potentiation of anti-PD1 on individual immune subpopulations. In particular, effects of AA+anti-PD1 on immunosuppressive subsets such as regulatory T cells and MDSCs, as well as the phenotype of infiltrating macrophages need to be delineated. Our data highlight the limitations of using immunocompromised mouse models to comprehensively study the anticancer effects of AA.

Early-phase clinical trials have demonstrated that intravenous AA at doses of up to 1 to 1.25 g/kg is well tolerated with chemotherapy, reduces the toxicity of chemotherapy, and may have antitumor activity (17). However, to our knowledge, this is the first time the effect of high-dose AA was studied in combination with immunotherapy. Our data reported herein, now provide compelling preclinical rationale for testing combinations of high-dose AA and anti-PD1 agents in patients with aggressive lymphomas such as diffuse large B cell lymphoma and also provide justification for testing this promising combination in the preclinical setting in other malignancies. The data indicate that high-dose AA is perhaps one of the most promising agents to potentiate the anticancer effects of anti-PD1 immunotherapy.

## Materials and Methods

### Cell Lines and Reagents.

The A20 (mouse B cell lymphoma; ATCC) cell line was used to establish the syngeneic mouse tumor model. Human diffuse large B cell lymphoma cell lines used in this study were SU-DHL-6 (ATCC), OCI-Ly1 (DSMZ), OCI-Ly7 (DSMZ), and OCI-Ly3 (DSMZ). OCILy1 and OCI-Ly7 were maintained in Iscove’s modified Dulbecco’s medium (IMDM) supplemented with 20% serum, SU-DHL-6 in RPMI 1640 with 10% serum, OCI-Ly3 in RPMI 1640 with 20% serum, and A20 cells in RPMI 1640 with 10% serum and 0.5 mM 2-mercaptoethanol. CD8+ T cells were isolated from healthy donor peripheral blood by negative selection (Stemcell Technologies) and cultured in X-VIVO 15 medium (Lonza) with 5% fetal bovine serum (FBS) in the presence of anti-CD3/CD28 beads (Dynabeads; Fisher Scientific).

### In Vitro AA Treatment.

Immediately prior to in vitro treatment with high-dose l-ascorbic acid (50-81-7; Sigma-Aldrich), cells were exposed to catalase to quench free radicals as previously published (8, 9); 100 μg/mL catalase (Sigma) in 50 mM potassium phosphate was applied to all cells for 30 min, prior to treatment with or without 1 mM AA (Sigma) for 6 h. Both catalase and AA were prepared fresh for each experiment. After the 6-h treatment, cells were washed and resuspended in fresh medium. Cells were harvested for downstream analyses 18 to 24 h after treatment.

### Genomewide DNA Methylation Analysis Using the HELP Assay.

OCI**-**Ly1 cells were treated with control (catalase) or high-dose (1 mM) AA + catalase for 6 h followed by incubation with fresh media for 18 h. DNA was extracted as per the protocol. Intact DNA of high molecular weight was corroborated by electrophoresis on 1% agarose gel in all cases. One microgram of genomic DNA was digested overnight with either HpaII or MspI (NEB). The following day the reactions were extracted once with phenol–chloroform and resuspended in 11 μL of 10 mM Tris⋅HCl, pH 8.0. The digested DNA was used to set up an overnight ligation of the HpaII adapter using T4 DNA ligase. The adapter-ligated DNA was used to carry out the PCR amplification of the HpaII- and MspI-digested DNA as previously described. Both amplified fractions were submitted to Roche-NimbleGen, Inc. for labeling and hybridization onto a human hg18 custom-designed oligonucleotide array (50 mers) covering 1.3 million HpaII amplifiable fragments (HAF). HELP microarray data have been deposited in the GEO database for public access (accession number GSE141397) (46). All microarray hybridizations were subjected to extensive quality control. Uniformity of hybridization was evaluated using a modified version of a previously published algorithm adapted for the NimbleGen platform, and any hybridization with strong regional artifacts was discarded. Bioinformatic analysis was done as previously reported (47).

### RNA Sequencing and HERV Analysis.

RNA sequencing was performed as previously described (38). The chromosomal positions (start/stop) of the HERVs were obtained from the HERVd (48) HG19 database, which we used to count the number of reads aligned to each locus. A differential expression analysis was performed on these HERV counts using the DESEQ2 (49) R package under default parameters. After annotating loci with Bioconductor’s ACME (50) package, we selected HERVs that had a *P* value <0.01 for the heat-map display, dividing each row by its mean to show normalized expression values and ordering along the *y* axis by groupwise fold change.

### Quantitative PCR.

RNA was extracted from frozen cell pellets using Qiagen RNeasy kit and reverse-transcribed to cDNA using SuperScriptIII cDNA (Invitrogen). Quantitative PCR for *CD274* and *GAPDH* was performed using Taqman primer-probe sets (Hs00204257_m1 and Hs02758991_g1; Life Sciences) and TaqMan Fast Advanced Master Mix (Life Sciences). Relative expression of *CD274* was calculated using the ΔΔCt method and represented as log_2_ fold change relative to the control group. Each reaction was performed in triplicate.

### LDH Cytotoxicity Assay.

CD8+ T cells were isolated from healthy donor peripheral blood by negative selection (Stemcell Technologies) and cultured in X-VIVO medium (Lonza) with 5% FBS and 1% penicillin/streptomycin in the presence of anti-CD3/CD28 beads (Dynabeads Human T-Activator CD3/CD28; Gibco/Thermo Fisher Scientific). CD8+ T cell enrichment was validated by flow cytometry. Two days after isolation, CD8+ T cells were treated with 100 μg/mL catalase for 30 min then divided for treatment with or without 1 mM AA for 6 h. CD8+ T cells (effector) were then removed from CD3/CD28 beads, resuspended in fresh medium, and combined with OCI-Ly1 cells (target) at a 1:1 effector:target ratio. Each condition was plated in triplicate in a 96-well plate each with 5,000 effector cells per well. Cocultured cells were incubated for 24 h and cytotoxicity was measured using the Pierce LDH Cytotoxicity Assay kit (Thermo Fisher Scientific) according to manufacturer’s instructions. Two independent biological replicates from different T cell donors were performed, each with 3 technical replicates. Percent cytotoxicity was calculated by the following equation:

$$\frac{\text{Experimental}\;\text{value}-\text{Effector}\;\text{cell}\;\text{spontaneous}\;\text{control}-\text{Target}\;\text{cell}\;\text{spontaneous}\;\text{control}}{\text{Target}\;\text{cell}\;\text{maximum}\;\text{control}-\text{Target}\;\text{cell}\;\text{spontaneous}\;\text{control}}\;\times 100.$$

### Cell Viability.

Cells were incubated at varying concentrations and time periods with l-ascorbic acid (50-81-7; Sigma-Aldrich) with catalase pretreatment at 100 μg/mL (9001-05-2; Sigma-Aldrich). Viability was assessed by the addition of Alamar Blue reagent (Thermo Fischer Scientific) and measured via Fluostar Omega Microplate reader. We found that antioxidant drugs interfere with cell viability measurements by assays that rely on the reducing property of viable cells. They directly reduce the reagent substrate to the reduced fluorescent form, giving spurious results. The protocol modification to counter this interference has been described and was used in this study (51).

### Flow Cytometry Cytotoxicity and Immunogenicity.

CD8+ T cells were isolated from healthy donor peripheral blood and were cultured in X-VIVO medium in the presence of CD3/CD28 beads for 48 h. After 48 h, the CD8+ T or OCI-Ly1 cells were treated with 100 μg/mL catalase or 30 min followed by 0 or 1 mM AA for 6 h. After 6 h, anti-CD/CD28 beads were removed from and T cells or OCI-Ly1 cells were washed and suspended in fresh medium. The CD8+ T cells and OCI-Ly1 cells were plated either alone or at the following ratios of effector:target cells: 1:1, 5:1, and 10:1. Cells were cultured for 48 h and CD8 (555366; BD Biosciences) expression was assessed by flow cytometry. Cytotoxicity was measured by 7-AAD (Sigma Aldrich) positivity and assessed in CD8+ (T cells) and CD8− (B cell) populations. Flow cytometry was performed on a BD Accuri flow cytometer.

### Syngeneic A20 Tumor Model.

All mouse experiments were approved by the Mayo Clinic Institutional Animal Care and Use Committee. BALB/c female mice (5 to 7 wk old) were purchased from The Jackson Laboratory. After a 1-wk acclimation period, 40 BALB/c mice received a subcutaneous (s.c.) right flank injection of 5 × 10^6^ A20 tumor cells in phosphate-buffered saline (PBS) and were randomly assigned to 4 groups of 10 mice: vehicle, anti-PD1 (α-PD1), AA, and AA+α-PD1. On day 9, palpable tumors were observed in 7 to 9 mice in each group (vehicle, *n* = 7; α-PD1, *n* = 9; AA, *n* = 9; AA+α-PD1, *n* = 8). Daily treatment was administered from day 10 until the tumor size endpoint was met (day 19). Mice in AA and AA+α-PD1 groups were given daily intraperitoneal (i.p.) injections of sodium l-ascorbate (Sigma-Aldrich). To balance the osmotic effect of sodium l-ascorbate, mice not receiving ascorbate (vehicle and α-PD1) were given NaCl (Sigma-Aldrich). To allow mice weighing ∼20 g to acclimate to the osmotic load, 1.5 M solutions of ascorbate or NaCl were first given at doses of 150 μL followed by 200 μL, each for 2 d. After this, 300-μL injections of 1.5 M ascorbate or NaCl were given daily. Added to these i.p. injections every other day was 200 μg anti-PD1 (BE0146; BioXCell) (α-PD1 and AA+α-PD1 groups) or isotype control (BE0089; BioXCell) (vehicle and AA groups) beginning on the first day of treatment. Tumor volume (cubic millimeters) was monitored every 2 d over the duration of the study by caliper and calculated as (Width^2^ × Length)/2, where W and L represent the shorter and longer diameters (millimeters), respectively. Significance between the growth curves was compared using the Student’s *t* test. Treatment continued until the first mouse reached a tumor volume of 2,000 mm^3^ (humane endpoint). Given the highly aggressive nature of the A20 lymphoma mouse model, humane endpoint was reached in one mouse in the vehicle group on day 19 (after only 9 d of treatment). To facilitate comparison between the treatment groups, all mice in the 4 groups were killed on day 19 by CO_2_ and tumors excised and weighed. Tumor weights between the treatment groups were compared using the Kolmogorov–Smirnov test of normality, Grubbs outlier test, and single-sided Student’s *t* test. Tumors were divided and immediately fixed in formalin for immunofluorescence and frozen for DNA analysis. CDI was used to calculate synergy with the combination using mean tumor weight measurements:

CDI = AB/(A×B),

where AB is the ratio of the 2-drug combination group to the control group and A or B is the ratio of the single drug group to the control group. CDI < 1 indicates synergism, and especially CDI < 0.7 indicates a significantly synergistic effect; CDI = 1 indicates additivity, and CDI > 1 indicates antagonism.

### Immunofluorescence.

Immunofluorescence and analysis was performed on formalin-fixed mouse tissues (*n* = 5 per treatment) by Reveal Biosciences. Each sample was processed and embedded in its own cassette and paraffin-embedded blocks were sectioned at 4 μm onto positively charged slides. Heat-induced antigen retrieval was performed using Leica Bond Epitope Retrieval Buffer 2 (ethylenediaminetetraacetic acid solution, pH 9.0) for 20 min. Nonspecific background was blocked with Novocastra Protein Block (RE7102-CE ; Leica) for 20 min. Each tissue was stained with primary antibodies against CD3, CD8, granzyme B, F4/80, CD11c, and PDL1 (Table 1) for 60 min. Secondary antibodies, goat anti-rabbit IgG Alexa Fluor Plus 647 (A32733; Thermo Fisher) or goat anti-rat IgG Alexa Fluor 647 (A21247; Thermo Fisher), were applied for 30 min. Slides were mounted with DAPI in Fluorogel II for nuclear visualization. Whole slide images were generated in fluorescence using a Pannoramic SCAN (3D Histech). Positive cells and intensity were quantified using AI technology ImageDx (Reveal Biosciences). All tissue and staining artifacts were digitally excluded from quantification. Data are reported as percent positive cells and as intensity.

**Table 1.** Immunofluorescence antibody staining conditions

Antibody	Supplier/catalog no.	Dilution	Host	Channel
Cd11c	Biorbyt/orb13554	1:100	Rabbit	Cy5
F4/80	Abcam/ab111101	1:400	Rabbit	Cy5
Granzyme B	Novus Biologicals/NB100-684	1:200	Rabbit	Cy5
PD-L1	Cell Signaling/13684	1:250	Rabbit	Cy5
CD8	Thermo Fisher/14-0808-82	1:200	Rat	Cy5/TRITC (costain)
IL-12	Abcam/ab131039	1:100	Rabbit	Cy5

### 5hmC MS/MS.

DNA was extracted from CD8+ T cells and mouse tumors (vehicle and AA groups only) using the Qiagen Blood and Tissue extraction kit. DNA was analyzed by MS for 5hmC at the University of North Carolina Biomarker Mass Spectrometry Facility. Data are expressed as 5hmC/10^6^ dC.

### Statistical Analysis.

One-way ANOVA and one-sided Student’s *t* test were used for statistical analyses as indicated. Statistical significance was considered *P* < 0.05.

## Supplementary Material

Supplementary File
