# Supplementary material for: High-dose ascorbic acid synergizes with anti-PD1 in a lymphoma mouse model
Source: Proc Natl Acad Sci U S A. 2020 Jan 7;117(3):1666–77. doi: 10.1073/pnas.1908158117 (PMC6983418; doi:10.1073/pnas.1908158117)
Supplement: Supplementary File [file pnas.1908158117.sapp.pdf]

SUPPLEMENTAL FIGURES

Figure S1.

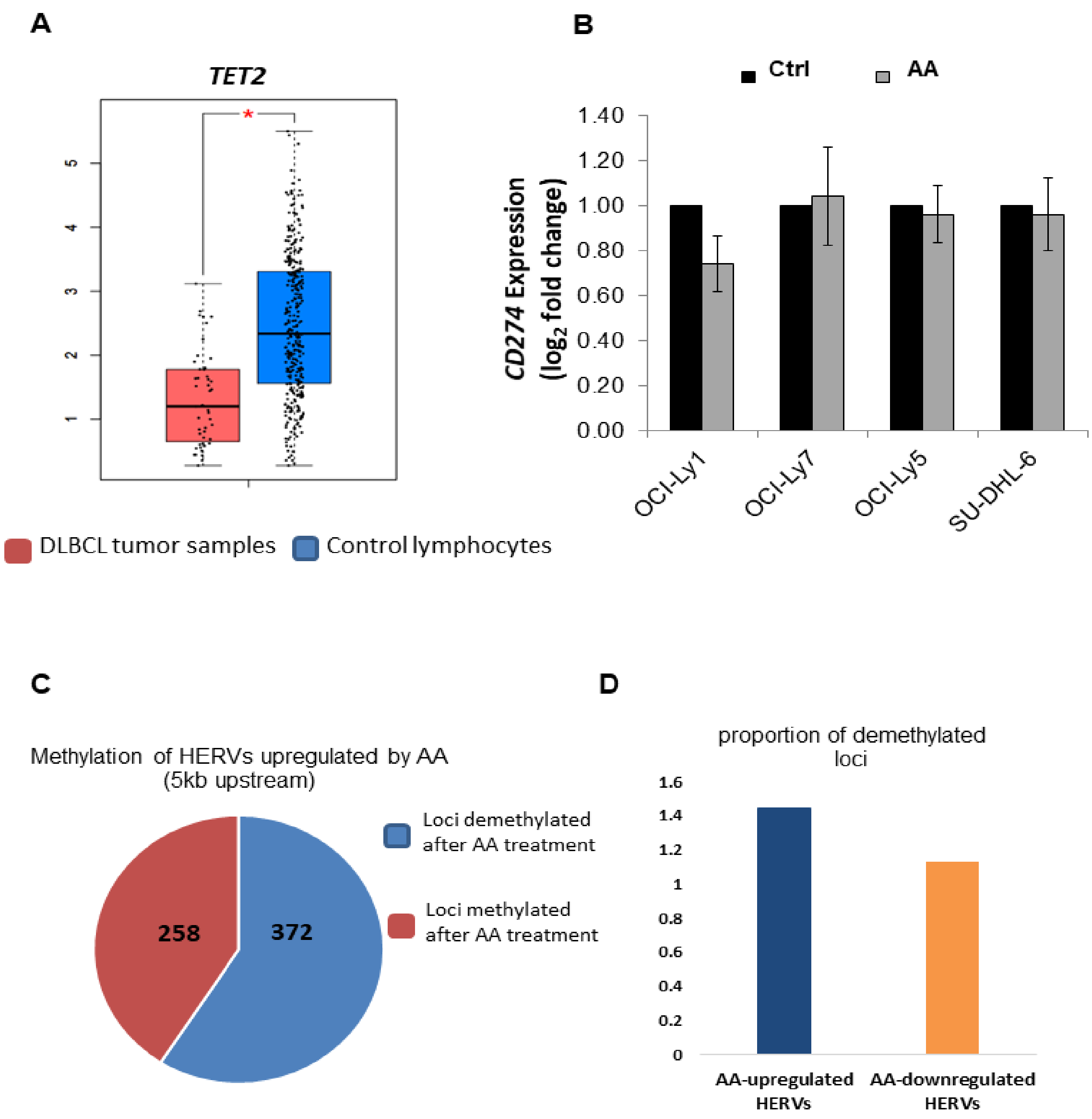

Figure S1. (A). TET2 mRNA expression in DLBCL (red) is significantly lower than control lymphocytes (blue) (P<0.05; TCGA, GTEX). (B). RT-PCR: PDL1 expression is unchanged after AA treatment in 4 DLBCL cell lines. (C). Global methylation analysis of HERVs upregulated with AA treatment revealed that ~60% of the loci (5kb window) were demethylated upon AA treatment. Loci representing both control treated (Ctrl_1, Ctrl_2) and AA treated (AA_1, AA_2) samples. (D) AA upregulated HERVs were associated with a higher proportion of demethylated loci when compared to the downregulated HERVSs (27% difference between AA-upregulated and downregulated HERV associated methylation). Data derived from both control and AA treated samples.

www.pnas.org/cgi/doi/10.1073/pnas.1908158117

Figure S2.

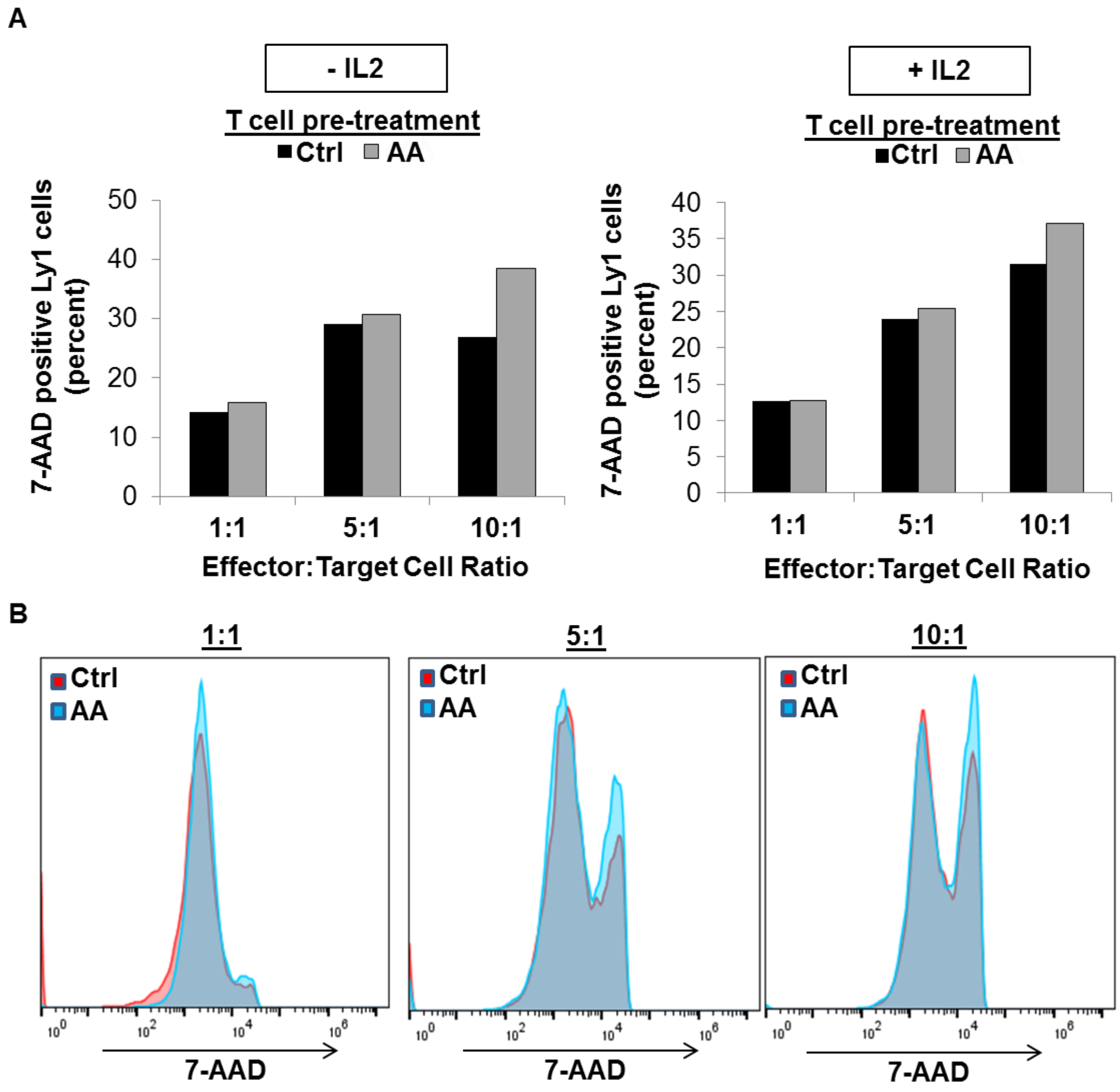

Figure S2. CD8+ T cells (effector cells) pre-treated with AA exhibit increased cytoxicity against OCI-Ly1 Bcell lymphoma cells (target cells).

(A). CD8+ T cells were pre-treated with catalase with or without 1 mM AA for 6 hours. Following treatment, cells were washed and incubated with OCI-Ly1 cells at the indicated ratios for 48 hours in the presence or absence of 10 ng/ml IL2 (B). Representative flow cytometry histograms.

Figure S3.

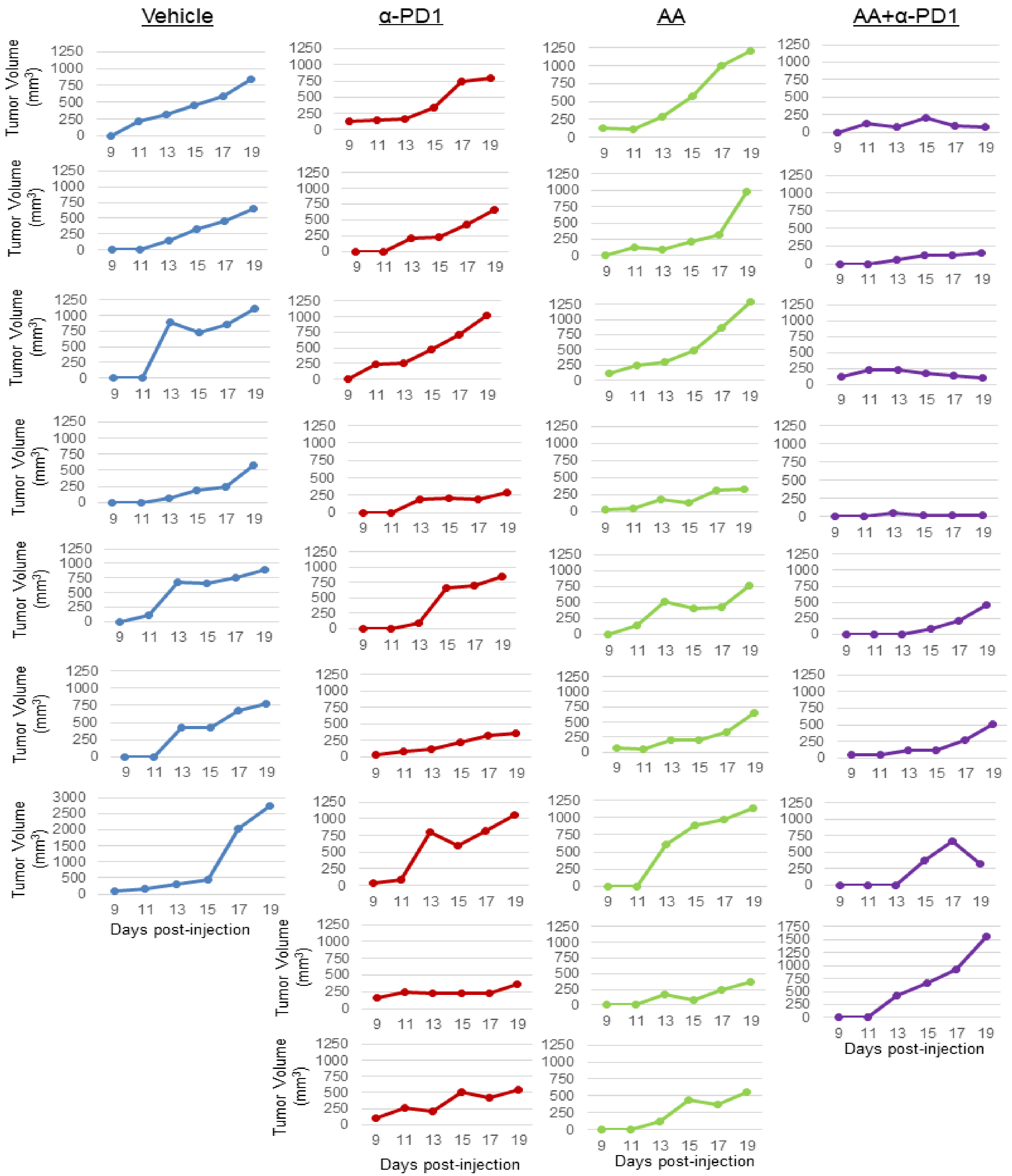

Figure S3: Growth curves of individual mice (Vehicle, n=7; α-PD1, n=9; AA, n=9; AA+α-PD1, n=8). All mice are represented in Fig 3B.

Figure S4: CD8 (cytotoxic T cells)

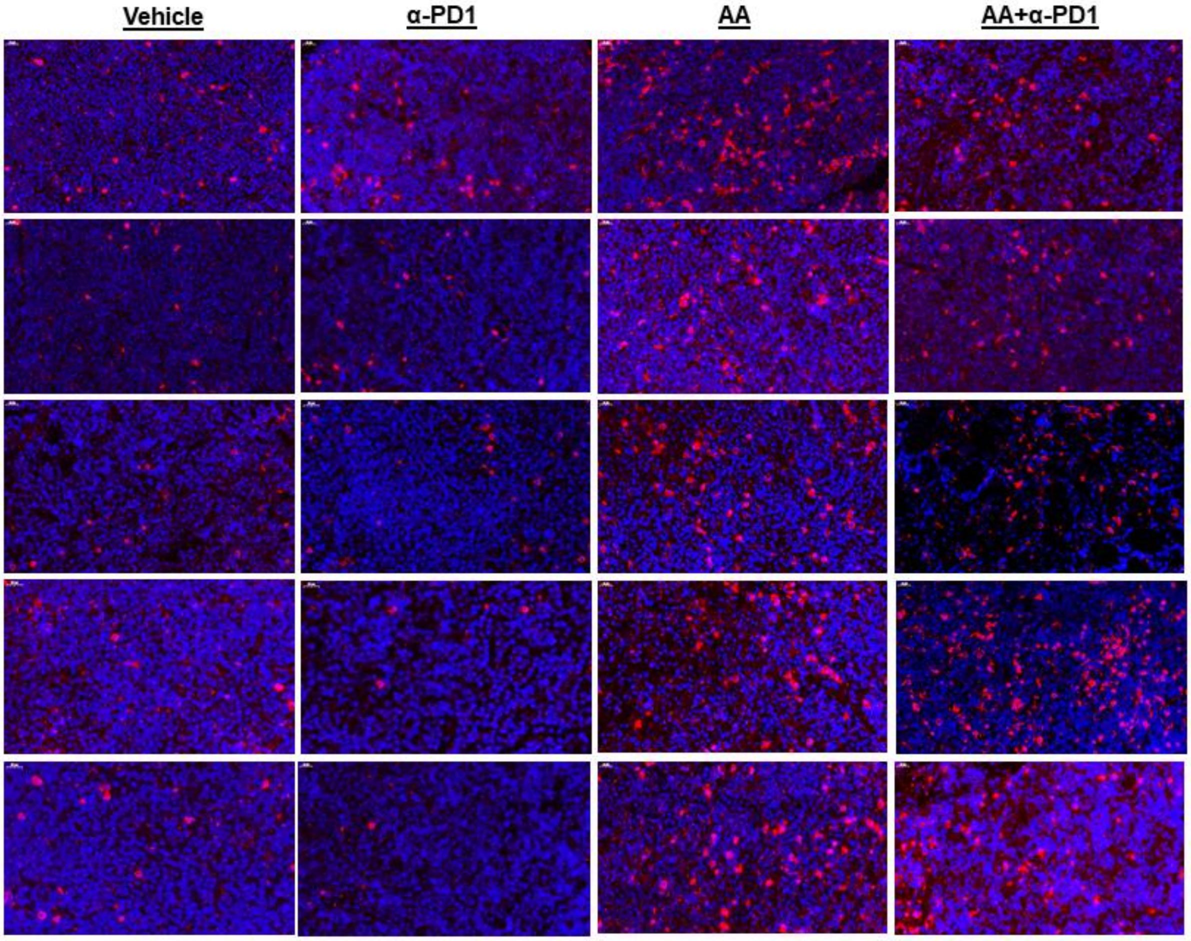

Figure S4. Representative CD8 immunofluorescence images for each mouse tumor tissue analyzed. α-PD1, anti-PD1; AA, ascorbic acid. Images were captured at 50x magnification.

Figure S5: Granzyme B

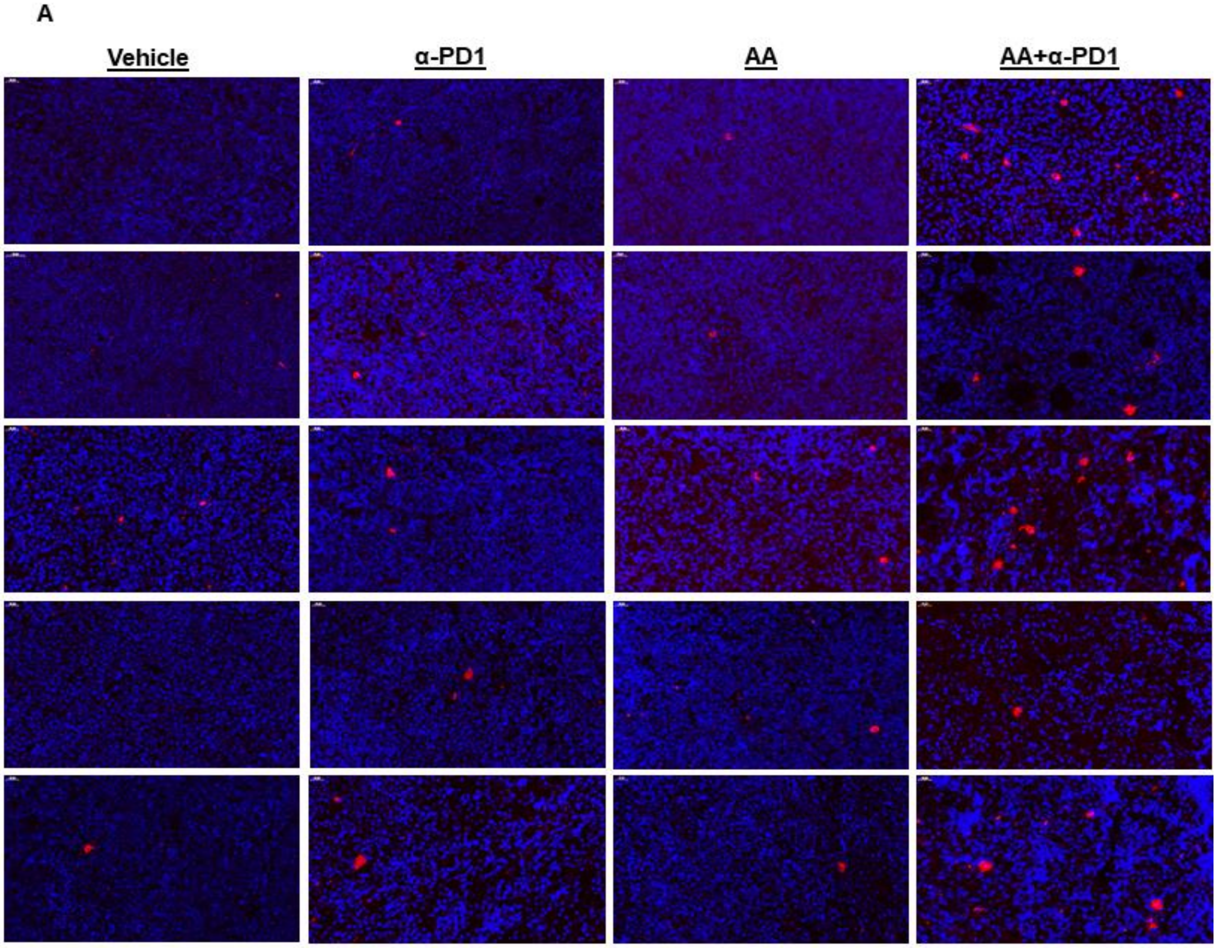

Figure S5. (A) Representative Granzyme B immunofluorescence images for each mouse tumor tissue analyzed. α-PD1, anti-PD1; AA, ascorbic acid. Images were captured at 50x magnification. (B) Granzyme B/ CD8 ratio is significantly higher with anti-PD1 treatment compared to AA treatment.

Figure S6: F4/80 (macrophages)

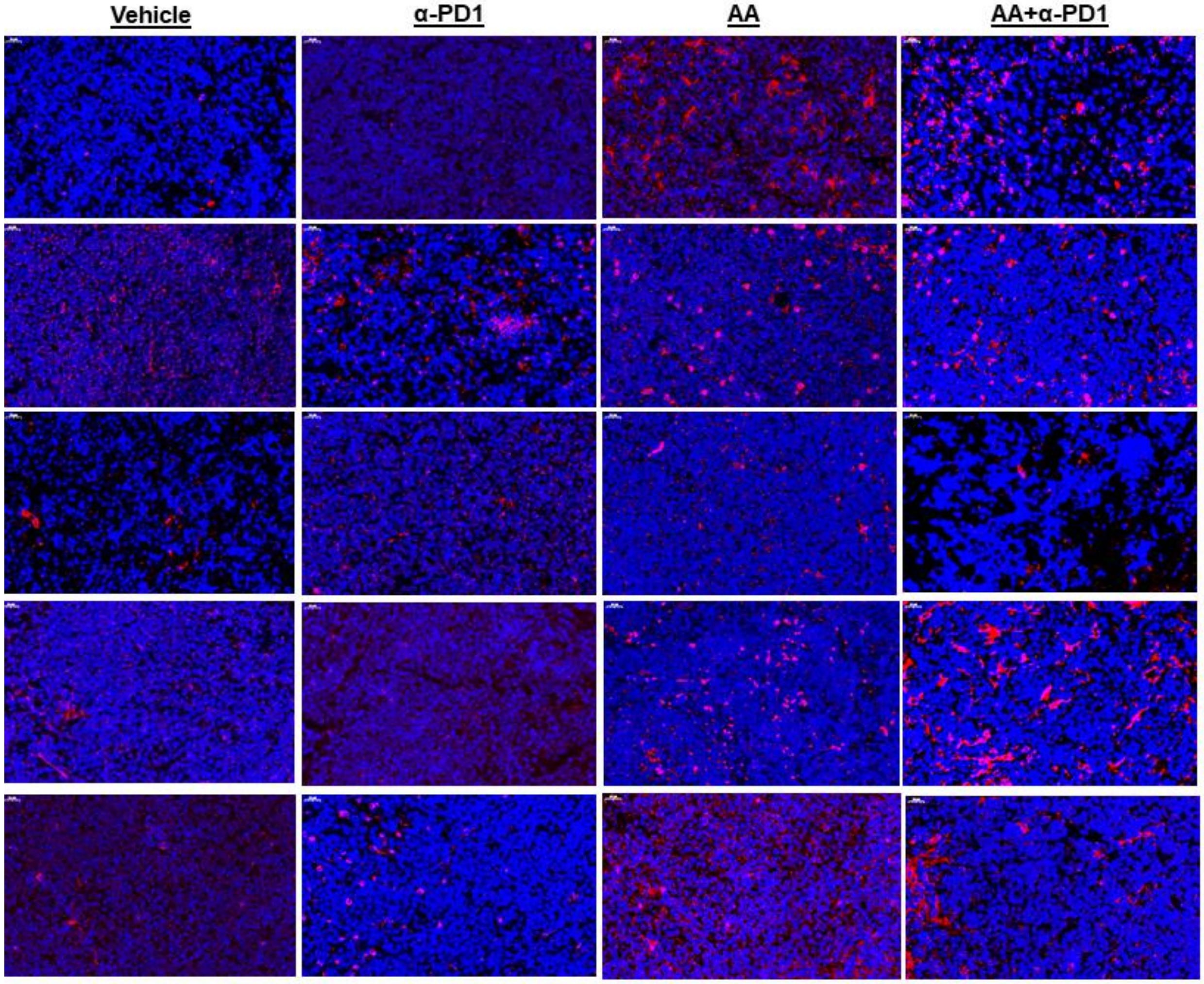

Figure S6. Representative F4/80 immunofluorescence images for each mouse tumor tissue analyzed. α-PD1, anti-PD1; AA, ascorbic acid. Images were captured at 50x magnification.

Figure S7: CD11c (dendritic cells)

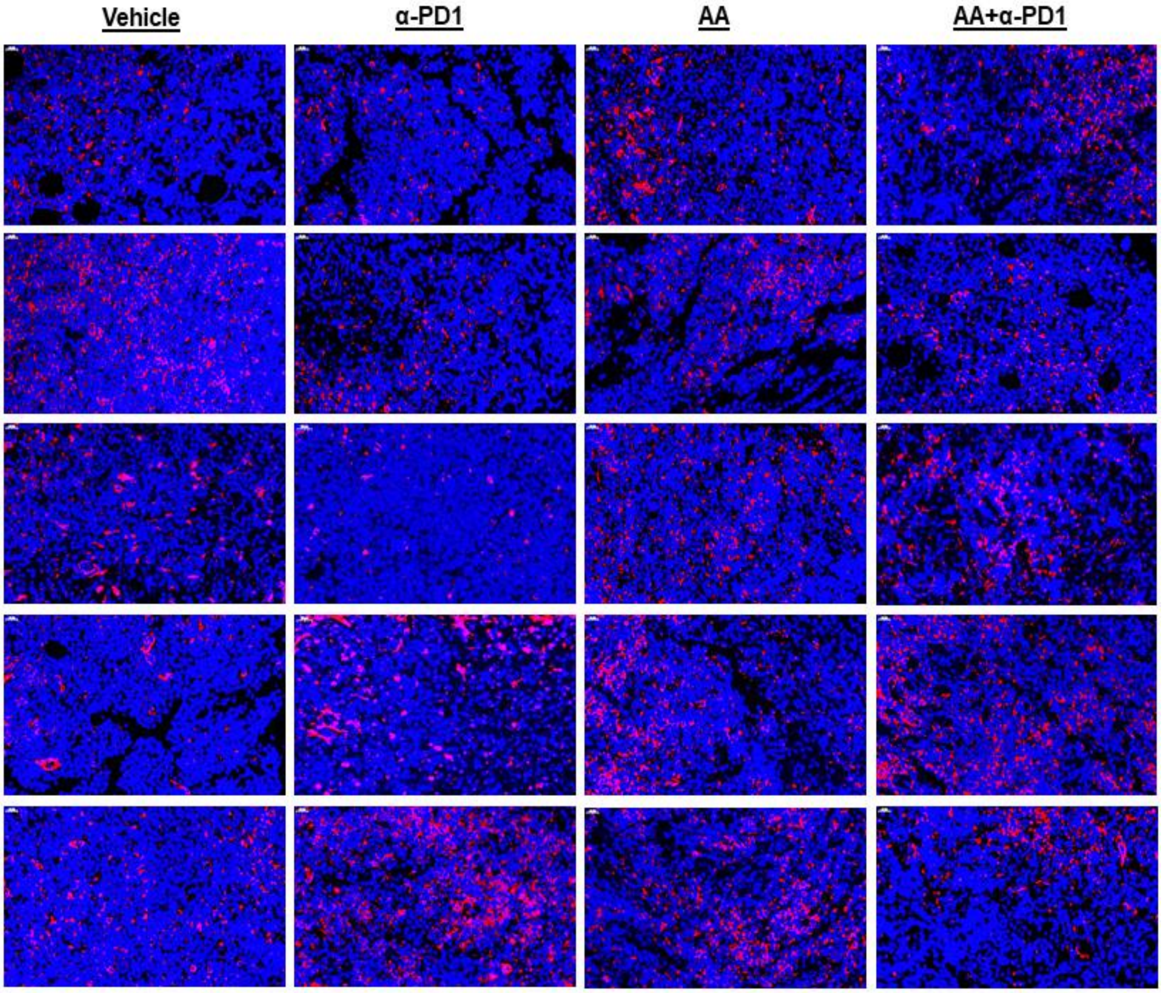

Figure S7. Representative CD11c immunofluorescence images for each mouse tumor tissue analyzed. α-PD1, anti-PD1; AA, ascorbic acid. Images were captured at 50x magnification.

Figure S8: PD-L1

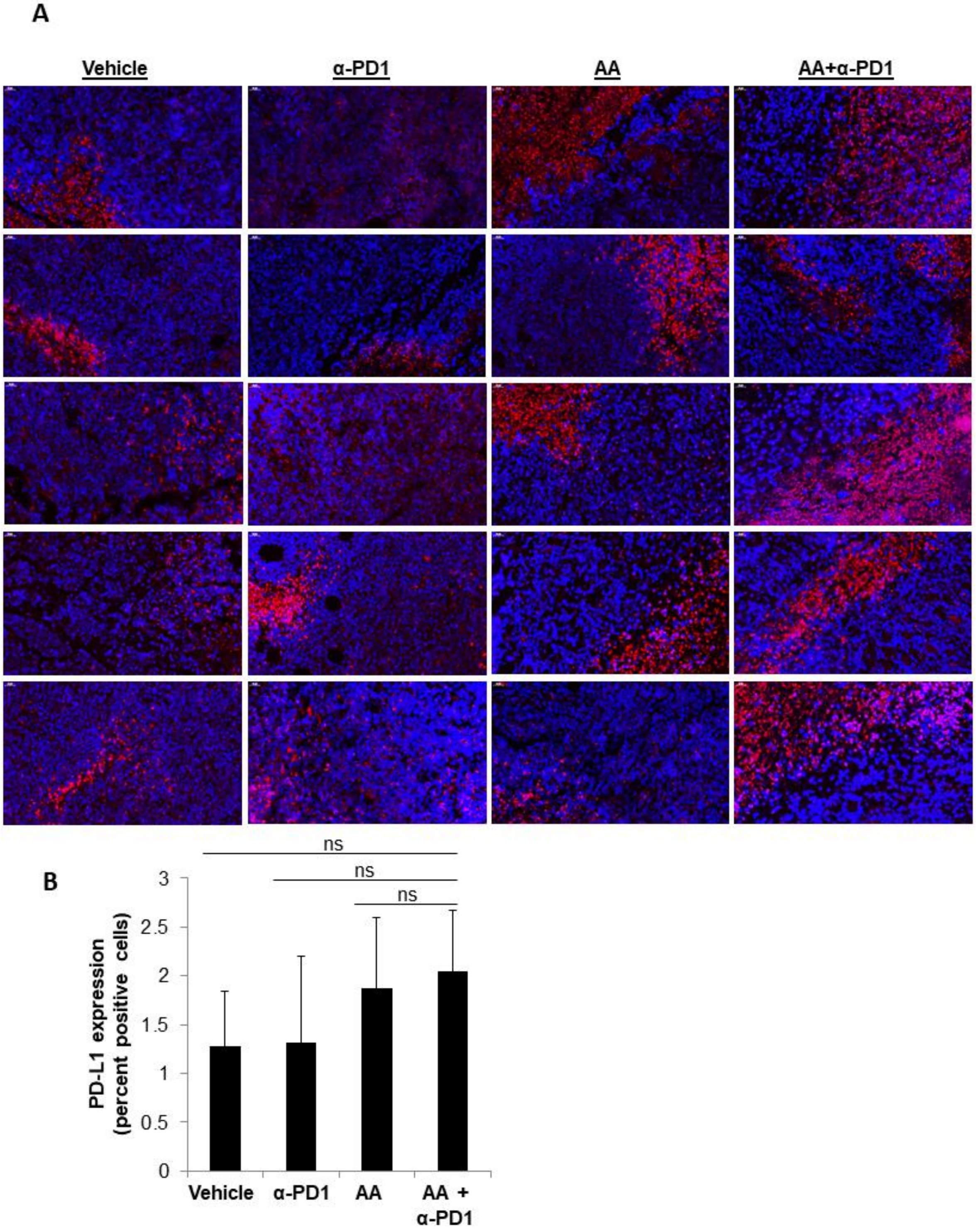

Figure S8. Representative PD-L1 immunofluorescence images for each mouse tumor tissue analyzed. α-PD1, anti-PD1; AA, ascorbic acid. Images were captured at 50x magnification.
